# Effects of Partially Filled EPS Foam on Compressive Behavior of Aluminum Hexagonal Honeycombs

**DOI:** 10.3390/ma17235945

**Published:** 2024-12-04

**Authors:** Itsara Rojana, Anchalee Manonukul, Julaluk Carmai

**Affiliations:** 1 The Sirindhorn International Thai-German Graduate School of Engineering (TGGS), King Mongkut’s University of Technology North Bangkok (KMUTNB), Pracharat 1 Road, Bangkok 10800, Thailand; 2 National Metal and Materials Technology Center (MTEC), National Science and Technology Development Agency (NSTDA), 114 Thailand Science Park, Pathum Thani 12120, Thailand

**Keywords:** honeycomb, EPS foam filler, filled structure, crushing characteristics, out-of-plane compression properties

## Abstract

This study investigates the compressive behavior of aluminum honeycombs partially filled with expanded polystyrene (EPS) foam, emphasizing the effects of filler area fractions and vertex contact locations on energy absorption and crush characteristics. Axial quasi-static compression tests evaluated energy absorption, mean crush force, specific energy absorption, and crush force efficiency. Results revealed that partially filled honeycombs significantly enhance energy absorption and mean crush force compared to their unfilled counterparts. However, higher filler area fractions increased mass, reducing specific energy absorption. Circular fillers exhibited lower energy absorption than hexagonal fillers due to their larger contact radius, which reduces stress concentration. The interaction between cell walls and fillers influenced densification strain, with wall–midpoint vertex contacts increasing peak force by reinforcing walls, while corner contacts reduced peak force but improved crush force efficiency. These findings underscore the potential of optimized, partially filled honeycombs for lightweight, energy-absorbing applications, particularly in automotive engineering.

## 1. Introduction

Metal honeycomb structures have long been valued in crashworthiness applications due to their superior energy absorption, strength-to-weight ratio, and resilience under impact loads [1,2]. This makes them ideal for aerospace, automotive, and high-speed train applications, where lightweight yet durable materials are crucial [3,4,5]. Recent studies have examined the crush behavior of honeycomb structures through analytical modeling [6,7,8], numerical simulations [9,10,11], and experimental testing [12,13,14] under various loading conditions [15,16,17]. Various honeycomb-based structures have been proposed as advanced composite materials, including functionally graded honeycombs [18], reinforced honeycombs [19,20], filled tandem honeycombs [21,22], bio-inspired designs [23], and honeycomb-filled structures [24,25]. A promising approach in this area involves filling thin-walled hollow structures with low-density materials like foams, which provide additional support against buckling and enhance crush stability. The early study investigated the filling of thin-walled columns with low-density materials, such as polyurethane (PU) foam [26], and found that PU foam fillers in cylindrical tubes reduced irregular buckling and promoted more symmetric and stable buckling patterns. Subsequent work examined the effects of quasi-static and dynamic loads on the behavior of foam-filled structures. An analytical solution using this experimental result was also developed to extend the scope and enable predictions for other geometries [27]. Notably, the interaction between foam and the tube wall significantly reduced folding length while increasing the number of lobes and the overall crushing force [28,29].

This study compares foam-filled circular and square tubes [30]. The effects of different foam filler densities in circular thin-walled tubes were also studied [31]. Additionally, the use of Polyethylene Terephthalate (PET) foam [32], aluminum foam [33], glass-fiber-reinforced polyamide [34], and honeycombs [35] as fillers in hollow, thin-walled structures has been widely investigated. Results showed that the interaction between the filler and structural wall significantly increased the mean crushing strength, yielding higher values than the combined strengths of the foam and structure alone when compared to unfilled thin-walled tubes.

Following the concept of filling thin-walled structures with effective materials, several studies filled the aluminum honeycombs with different materials. The results indicated an increase in mean crushing strength and energy absorption capacity, with foam filling having a greater effect on honeycombs with lower densities [36]. Studies on aluminum honeycombs filled with expanded polypropylene (EPP) foam further examined the effects of filler density. Findings showed that total energy absorption increased with higher foam density, while specific energy absorption remained unchanged [37]. The effect of strain rate on the mechanical response of PU foam-filled aluminum honeycombs was also investigated [38]. Investigations on aluminum honeycombs filled with different types of EPP foam demonstrated that initial peak and mean strength increased due to the interaction between cell walls and foam, although specific energy absorption decreased. Comparisons of different filling types concluded that single-cell filling was most effective for enhancing load capacity with minimal filling [39]. In addition, the study of foam concrete filling in aluminum honeycombs showed that although foam concrete is brittle, reducing the cell size in honeycombs with foam filling improved load bearing and energy absorption capacity compared to unfilled honeycombs [40].

Although fully filled aluminum honeycombs enhance mechanical properties, the overall mass also increases and potentially diminishes specific energy absorption (*SEA*)—a key parameter in automotive applications where weight minimization is essential. To address this, partially filled honeycomb structures have gained attention as a compromise, offering significant specific energy absorption benefits. Nevertheless, limited research has focused on the effects of partial filling configurations, particularly how filler area fractions and vertex contact locations influence crush performance.

This study aims to examine the compressive behavior of aluminum honeycombs partially filled with expanded polystyrene (EPS) foam. By investigating different filler area fractions, filler shapes, and their vertex contact locations within honeycomb walls, this work seeks to clarify the role of partial filling in optimizing crush characteristics—especially for applications requiring both weight efficiency and high energy absorption. A comprehensive experimental investigation was conducted to compare the out-of-plane compressive behavior of bare and partially filled honeycombs, varying filler area fraction, and vertex location. EPS foam fillers with six different area fractions were examined, with filler vertices located either at the midpoint of honeycomb cell walls or at cell corners. The influence of filler area fraction and filler contact location on mechanical responses and crush characteristics was analyzed and discussed. 

## 2. Materials and Methods 

### 2.1. Materials and Specimen Preparation

**Figure 1:**
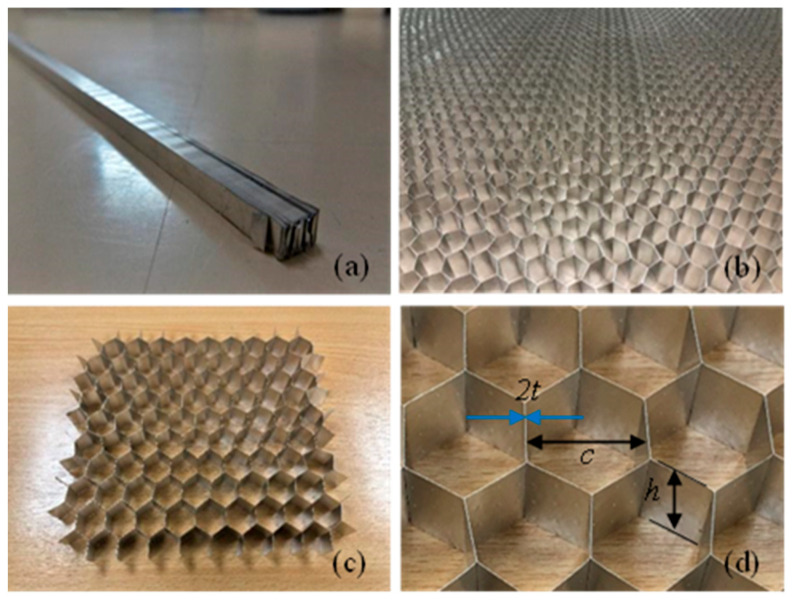
Aluminum alloy hexagonal honeycombs: (**a**) unexpanded honeycombs, (**b**) expanded honeycombs, (**c**) cut specimens of bare honeycombs, and (**d**) the geometry of a representative unit cell of honeycombs.

The commercially available hexagonal cell honeycombs (Corex Honeycomb, Huntingdon, United Kingdom) were used in the present study. The honeycombs were made of aluminum alloy grade 3003 and were supplied in an unexpanded form as shown in Figure 1a. Unexpanded aluminum honeycomb structures were manually stretched until reaching the desirable hexagonal shape (Figure 1b). The honeycomb compression specimens were carefully cut to preserve the cell wall and maintain the original cellular structure. To ensure accurate results, specimens with a 9 × 9 cell configuration are employed (Figure 1c). Specimens containing a smaller number of cells were also tested, but inconsistent and bad representative compression results were obtained. This is also observed elsewhere [15]. Figure 1d shows the geometry of a representative unit cell with the cell size (*c*) of 19.1 mm, the constant foil thickness (*t*) of 0.01 mm, and the honeycomb height (*h*) of 20 mm. Two of the six cell walls in the representative unit cell possess double thickness, as indicated by the blue arrows in Figure 1. The double-thickness wall, denoted as 2t in Figure 1d, forms at the bonded area where two adjacent walls overlap and are glued together during the fabrication process.

**Figure 2:**
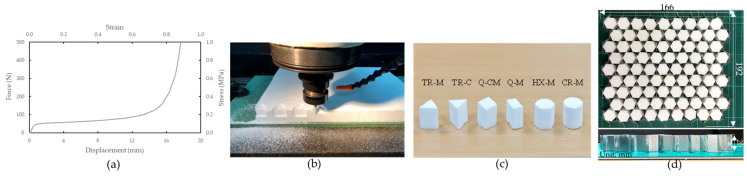
(**a**) Stress–strain curve of EPS. (**b**) Preparation of EPS foams as internal fillers using CNC machining process (**c**) machined EPS fillers with different shapes, and (**d**) the overall dimension of the test specimen.

The EPS foam material (Asia Plastic Industry Co., Ltd., Bangkok, Thailand), with a density of 48 kg/m³, was used as reinforcement fillers. It is important to note that the EPS foam used in this study has not been treated with fire retardants nor tested for flame resistance as part of this research. As such, it may not meet specific fire safety standards. However, the foam could potentially be treated with flame-retardant additives or coatings to enhance its fire resistance for automotive applications [41]. The mechanical properties of the bulk EPS foam were determined by compressing a cylindrical sample with a diameter of 19 mm and a height of 20 mm at a velocity of 1.2 mm/min. The resulting stress–strain curve is presented in Figure 2a. Due to the rigidity and toughness of EPS foams, directly punching them into the desirable filler shapes proved impractical. The EPS foam sheet was machined using a computerized numerical control (CNC) machine (Figure 2b) to produce six distinct filler shapes, as shown in Figure 2c. The dimensions of each filler shape were pre-calculated to precisely fit the 19.1 mm honeycomb cells. These dimensions and shapes were specified in a CAD file and uploaded to the CNC program. The cutting tool’s feed rate and spindle speed were experimentally adjusted to ensure accurate fillers that fit the honeycomb cells precisely. The forming process with a feed rate of 0.5 mm/s and a spindle speed of 2000 rpm was optimized to produce high-quality, defect-free filler surfaces. Foam fillers were manually inserted into the hollow honeycomb cells and adjusted to ensure tight contact, utilizing a non-adhesive interface between the fillers and the cell walls. Before testing, the fillers were visually inspected and adjusted to ensure proper alignment. The cross-sectional shapes of the fillers included triangle (TR), quadrilateral (Q), hexagonal (HX), and circular (CR). The overall dimensions of each test specimen were 166 mm × 192 mm × 20 mm (width × length × height), as shown in Figure 2d.

**Figure 3:**
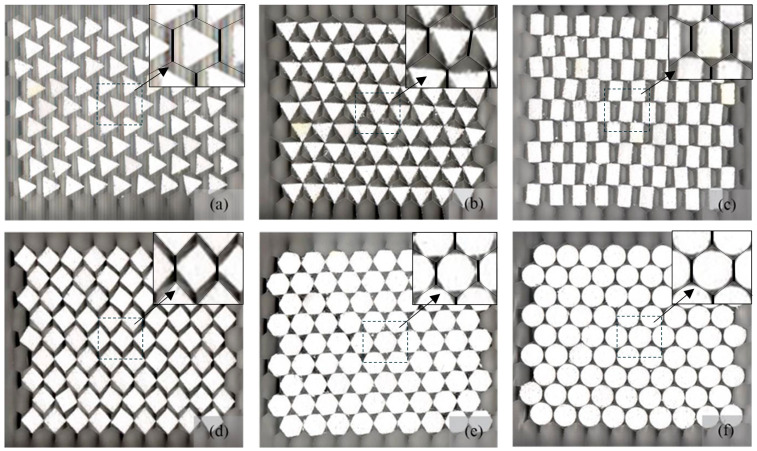
EPS foam partially filled Al honeycombs with different shapes: (**a**) triangular filler with wall–midpoint reinforcement (TR-M), (**b**) triangular filler with corner reinforcement (TR-C), (**c**) rectangular filler with wall–midpoint reinforcement (Q-M), (**d**) rectangular filler with corner and wall–midpoint reinforcement (Q-CM), (**e**) hexagonal filler with wall–midpoint reinforcement (HX-M), and (**f**) circular filler with wall–midpoint reinforcement (CR-M).

The orientation of the triangular and quadrilateral foam filler shapes within the hexagonal cells resulted in two different area fractions, while the orientation of the hexagonal and circular shapes did not affect the area fraction, as shown in Figure 3. The orientation of both the triangular and quadrilateral shapes also resulted in different positions for the filler vertices: the vertices could be located at the midpoint of the cell walls, denoted as M, or at the corners of the honeycomb cells, denoted as C. As a result, each EPS foam partially filled into the aluminum honeycomb compression specimen had a designated name in the format [cross-section of filler]-[location of the vertices] throughout this study. For example, TR-M referred to the triangular EPS foam fillers with the vertices located at the midpoint of the honeycomb cell walls, as shown in Figure 3a. Additionally, the bare aluminum alloy honeycomb compression specimen designated as H19 was also tested as a reference. Figure 3 shows all six specimens with different foam filler shapes and area fractions used in this study. The vertices of fillers TR-M (Figure 3a), Q-M (Figure 3d), HX-M (Figure 3e), and CR-M (Figure 3f) specimens were located at the midpoint of honeycomb cell walls. The vertices of the TR-C fillers (Figure 3b) are located at the corners, and Q-CM (Figure 3c) are designed to compare with the corresponding filler shapes supported at the cell walls, i.e., TR-M and Q-M, respectively. TR-C (Figure 3b) was the honeycomb filled with triangular foam fillers, which enhanced the honeycomb strength by supporting at three corners. The honeycomb specimens are filled with rectangular fillers (Q-CM, Figure 3c), reinforcing honeycomb cells at two corners and two double-thickness walls. However, TR-C and Q-CM had slightly higher area fractions than the corresponding shape reinforced at the cell walls (TR-M and Q-M) due to the geometrical constraint of filler–wall contact, that the fillers were required to touch the honeycomb walls. Table 1 summarizes all six types of specimens with their corresponding area fraction used in this study. 

### 2.2. Experimental Setup

**Figure 4:**
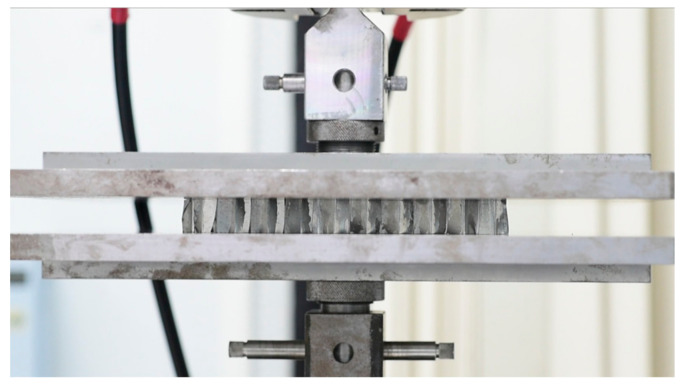
The specimen was clamped between movable upper and fixed lower platens in a universal testing machine.

Mechanical properties of EPS foam with partially filled honeycombs were tested at room temperature using a 50 kN HST WDW-50E universal testing machine (Jinan Hensgrand Instrument Co., Ltd, Jinan, China). The specimen was clamped between two platens of the size 220 × 200 mm, as shown in Figure 4. It was centered manually. The upper platen was moved down to the position where it was about to contact the specimen, and this position was recorded as zero. All foam-filled honeycomb specimens without face sheet panels were subjected to the axial quasi-static compression tests using a constant compression rate of 1.2 mm/min with a maximum compression force of 30 kN. During compression, a data acquisition unit continuously recorded the load and displacement data with a sampling rate of 10 Hz. The ratios of compressive force to initial cross-section area and crush displacement to initial thickness of the specimen were defined as nominal stress and nominal strain, respectively, without considering the effect of geometric discontinuities and displacement. A minimum of three tests were performed for each sample set, and the mean values were determined.

### 2.3. Crushing Characteristics

A variety of crushing indicators can be employed to assess the crashworthiness performance of the honeycomb structure. The initial buckling of honeycomb walls was concomitant with the occurrence of the first maximum peak force (*P_max_*). It was desirable to achieve a low peak force and avoid secondary impact damage. Subsequent to the onset of buckling in the honeycomb wall, the structure absorbed energy through progressive wall collapse mechanisms. The capability of energy absorption is also dependent on the deformation mode and material. The energy absorption (*EA*) of compressed specimens is defined as the area under the load–displacement curve and can be calculated using Equation (1) [37].

(1) EA =∫Fds

where *EA* is the energy absorption; *F* is the instantaneous crush force; and *s* is the crush displacement. 

Weight reduction was a primary design objective in both the aerospace and automotive applications. In the automotive sector, lightweighting is particularly critical. To optimize the weight–energy absorption capacity, specific energy absorption (*SEA*) is a key performance metric. [42], defined as the ratio of absorbed energy (*EA*) to structural mass (*m*), as shown in Equation (2) [43].

(2) $$SEA=\frac{EA}{m}$$

The compressed structures continue to collapse until reaching the densification stage, where stress increases rapidly with small strain. The squeezed structure is almost without any energy absorption capability at a densification stage. The critical strain at this stage is the densification strain (*ɛ_d_*). The energy efficiency method was used to indicate *ɛ_d_*. Energy efficiency (*EFF*) is defined as the ratio of energy absorption depending on a particular strain divided by the stress itself, as shown in Equation (3) [44].

(3) $$EFF\;(\varepsilon )=\frac{1}{\sigma (\varepsilon )}\int \sigma (\varepsilon )d\varepsilon ,\;0\;\leq \;\varepsilon \;\leq \;1$$

whereas σ is the stress at the corresponding strain (ɛ).

The densification strain is where the energy efficiency reaches the maximum on the energy efficiency–strain curve (*ɛ_d_* = *ɛ*[*EFF*(*ɛ*)]_max_), which should satisfy the condition of maximum efficiency [15,45].

The mean crush force ($P_{m}$) is the ratio of the absorbed energy (*EA*) to the displacement (*S_d_*) at densification strain, and it can be calculated by Equation (4) [46].

(4) $$P_{m}=\frac{EA}{S_{d}}$$

Crush force efficiency (*CFE*) is defined as the ratio of the mean crush force (*P_m_*) to the maximum peak force (*P_max_*) and can be obtained by Equation (5) [47].

(5) $$CFE=\frac{P_{m}}{P_{max}}\times 100\%$$

## 3. Results and Discussion

### 3.1. Effect of EPS Foam Filling on Compressive Response 

**Figure 5:**
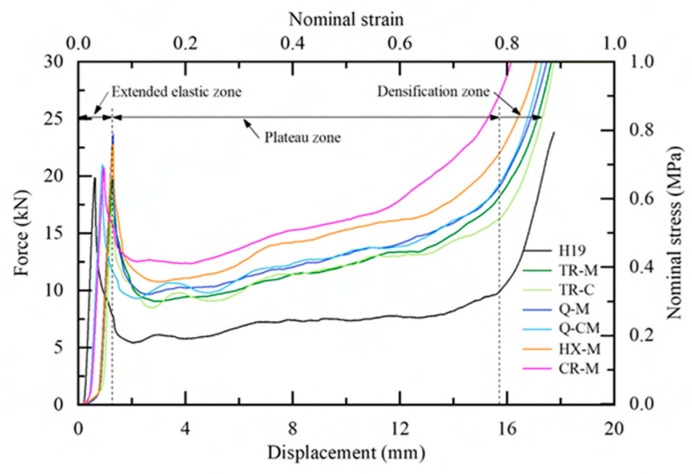
Representative force–displacement and nominal stress–strain curves of bare honeycomb and honeycombs filled with EPS foam fillers subjected to axial compressive loading under quasi-static conditions (for interpretation of the references to color in this figure legend, the reader is referred to the web version of this article).

The representative force–displacement curves for bare and EPS foam partially filled honeycombs, with various area fractions of foam filling, under quasi-static compression are shown in Figure 5. The response of the bare honeycomb specimen, serving as a reference, is represented by the black curve. All force–displacement curves shown in Figure 5 exhibit the same trend, which can be divided into three zones: the elastic zone, the plateau zone, and the densification zone [1,38].

For bare honeycombs (H19, black curve in Figure 5), force increases linearly with the crush displacement in the elastic zone and reaches an average peak force of 18.4 kN at the crush displacement of 0.52 mm. The maximum peak force (*P_max_*) represents the initial first buckling of the cell wall [1]. The force drops rapidly to 7.4 kN after the initial first buckling. The plateau zone then starts due to the progressive buckling and deforming of cell walls [48]. The crush force fluctuates around the plateau force of 7.4 kN (*P_m_*) as the crush displacement continues increasing until 15.8 mm. The force started to rise rapidly with increasing displacement from this crush displacement, defined as the densification displacement (*S_d_*). The response entered the densification zone, where there was no further collapse of the wall, and the material deformed permanently, the density of the specimen increased. 

Partially filled foam honeycombs exhibit similar force–displacement curves but different magnitudes and displacement values, as depicted in Figure 5. Table 2 summarizes the average crush characteristic indicators of all cases with various area fractions and shapes of foam-filled specimens. 

The force–displacement curves of partially filled honeycombs exhibited similar three-zone characteristics to those observed in the bare honeycombs (H19). An extended elastic zone was observed in all cases of partial foam filling compared to the bare honeycomb. This phenomenon is also consistent with the findings in [37] for fully filled honeycombs. The presence of EPS foam fillers demonstrably influenced the crush behavior in the elastic zone. This extended elastic zone exhibited two distinct sub-zones. The first sub-zone starts from the beginning. The force gradually increased with displacement. In the second sub-zone, the force started sharply and linearly increasing, while the crosshead was continuously compressed at a constant speed. Specimen-preparing imperfections resulting from foam cutting can be attributed to this observed behavior. The first sub-zone is likely resulting from gaps between the filler and the honeycomb wall. In the second sub-zone, all gaps were diminished, hence the rapid increase in compressive force.

Considering the cases where the vertices of the foam filler were located at the midpoint of the wall (filler–wall contact) with varying area fractions (TR-M, Q-M, HX-M, and CR-M), it is found that as the area fraction of EPS foam increases, the elastic zone tends to extend further. Both the peak force and the crush displacement at the peak force (*S_p_*) generally increased with higher EPS foam area fractions, except in the case of CR-M. The mean crush force showed a positive correlation with the area fraction of EPS foam. Additionally, an increase in the foam area fraction resulted in a reduction in densification displacement, except for CR-M. 

When comparing the peak force of the foam-filled honeycomb with the bare honeycomb, the differences ranged from 2.7% for the smallest foam area fraction to 34.8% for the largest foam area fraction. The difference in mean force varied from 37.8% for the smallest area fraction to 94.6% for the largest area fraction. The critical displacement, which corresponds to the onset of densification, was observed to decrease significantly in the foam-filled honeycombs, with a reduction ranging from 1.3% to 13.9% compared to the unfilled honeycomb structures, as summarized in Table 2. This is expected, as the load-carrying capacity increased with the area fraction of EPS fillers.

Comparing HX-M and CR-M (the orange and purple curves in Figure 5, respectively), CR-M (77.63%) has a slightly larger area fraction than HX-M (74.43%), and the fillers for both specimens are supported at all six-midpoint walls. The HX-M samples have significantly higher peak force, displacement at peak force, and densification displacement than the CR-M samples. However, the mean crush forces for both are similar. This is because the EPS fillers in CR-M samples are cylinders with a significantly larger radius, while the hexagonal fillers have corners with a small radius. This phenomenon is also consistent with the findings in [28,29] for comparison between filled circular and square filled tubes. The stress concentration from EPS fillers with a larger radius is likely to be lower. Hence, this significantly influences the initial elastic regime.

When comparing cases with the same foam filler shape but different foam area fractions and vertex contact locations—i.e., TR-M (wall–midpoint contact, Figure 3c) and TR-C (corner contact, Figure 3d)—it is found that the force–displacement curves (the dark green and light green curves in Figure 5, respectively) exhibit a similar trend, except for the peak force, as summarized in Table 2. Although the TR-C case had a 6.72% higher foam area fraction than TR-M, the peak force obtained from the TR-C case was 9.6% lower. Additionally, the displacement at the peak force and the critical displacement for TR-M were 16.9% and 14.7% lower, respectively, than those for TR-C.

This evidence suggests that the vertex contact location also influences the crush characteristics. When the vertices of the foam filler were located at the honeycomb cell wall, the peak force was higher, even though the area fraction was lower than in the case where the vertices were at the corner. Strategic placement of the filler vertices at the cell wall midpoint resists the initial buckling of the cell wall, thus increasing the peak force.

In addition, Q-M and Q-CM are characterized by identical quadrilateral filler shapes, but they differ in terms of filler vertex contact locations (Figure 3c,d). Despite a 9.7% lower foam area fraction, Q-M exhibited a 6.7% higher peak force than Q-CM, further highlighting the influence of vertex contact location on peak force. For Q-M, all four vertices of the foam filler were positioned at the midpoint of the cell walls, while in Q-CM, two vertices were in contact with the cell walls and two with the corners. The quantity of cell walls supported by the foam filler impacted the peak force as a wall-strengthened reinforcement. The filler exhibited resistance to collapse, requiring a higher force to induce crushing. The crushing responses within the plateau and densification zones were almost identical, characterized by similar critical displacements, as shown in Figure 5. 

Based on the axial quasi-static compression results in Figure 5, these findings suggest that the partially filled honeycomb structures with different area fractions and vertex contact locations have a significant influence on the extended elastic zone and the reduced critical displacement.

### 3.2. Effect of EPS Foam Fillers on Crush Characteristics

**Figure 6:**
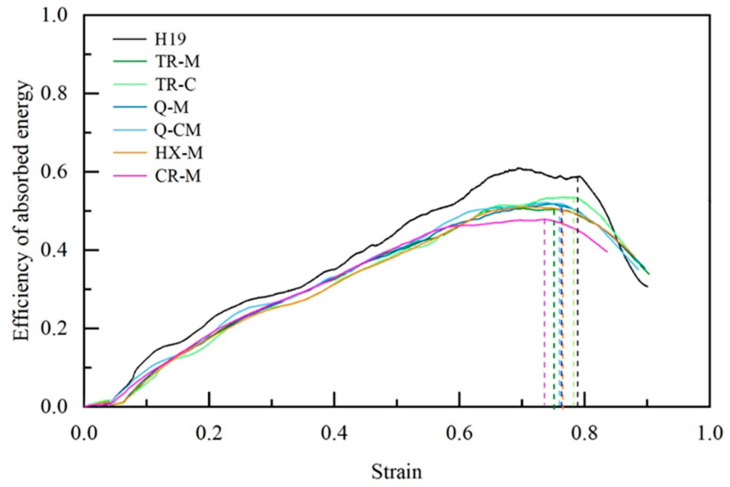
Efficiency of absorbed energy as a function of strain for aluminum honeycombs with various fillers.

As depicted in Figure 6, the absorbed energy efficiency curves for all tested specimens demonstrate comparable trends as a function of strain. The energy absorption efficiency increased with increasing strain, reaching a maximum point defined as the densification strain. Subsequently, efficiency decreased dramatically. This behavior corresponded to a sharp rise in the force response, indicating complete collapse of the deformed wall. 

The crushing characteristics of partially EPS-filled honeycomb structures were evaluated using densification strain, energy absorption, specific energy absorption, and crush force efficiency as indicators. All these indicators were calculated from the experimental results for all six cases stated in Table 1. Figure 6 illustrates the densification strain and measured mass of the specimen as a function of the area fraction of fillers. It was noted that the area fraction is calculated while the mass is measured. The height of the foam filler was almost the same; hence, the filler mass increased with the area fraction, as expected. Deviations from perfect linearity in the observed relationships can be attributed to variations in the specimens, such as non-uniform EPS foam density, dimensional inconsistencies caused by CNC machining of the foam, and irregularities along the edges of the aluminum honeycomb. The mass of the aluminum honeycomb filled with hexagonal EPS foam fillers exhibited the greatest variation. 

**Figure 7:**
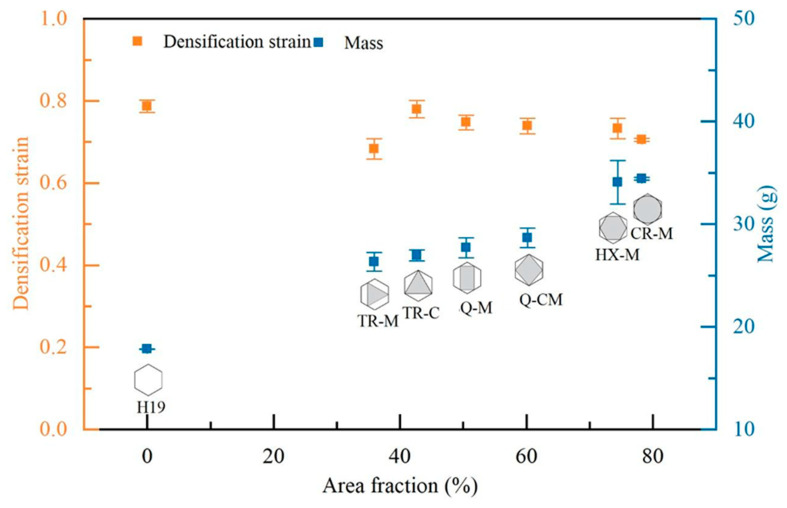
Densification strain and measured mass of specimen as a function of area fraction of fillers.

Comparing the densification strain calculated from the experimental results among TR-M, Q-M, HX-M, and CR-M, of which the vertices are located at the midpoint of the cell walls, as illustrated in Figure 7, all EPS-filled honeycombs displayed lower densification strains than that calculated from the bare honeycomb (H19). The CR-M case showed a lower densification strain than the others, but the area fraction of the foam filler was higher. The observed difference in stress concentration arises from the contrasting contact areas, with the CR-M configuration’s circular filler exhibiting larger contact areas compared to the smaller contact areas of the fillers in the other configurations.

In the TR-C case, where all three vertices of the foam filler were in contact with the three corners of the honeycomb cell, all six cell walls collapsed freely without resistance from the foam fillers at the beginning. This collapsing behavior closely resembles that of the bare honeycomb. The densification strain of the TR-C case was the highest among the foam-filled specimens and similar to that of the bare honeycomb. As the cell walls gradually collapsed, a small portion at the vertices of the EPS foam was penetrated by the folded honeycomb walls. In contrast, in the TR-M case, where all three vertices of the foam filler were in contact with the midpoint of the cell walls, the walls exhibited initial resistance to collapse due to the presence of filler reinforcement at the midpoint of the wall. During the compression test, the EPS foam was penetrated by the collapsed walls. The TR-M configuration exhibited a larger area of foam penetration compared to the TR-C configuration, resulting in a lower densification strain. This localized interaction behavior was explained in detail in Section 3.3.

It was also found that the densification strain of Q-CM was lower than that of Q-M despite the area fraction being higher, but the vertex locations were different.

**Figure 8:**
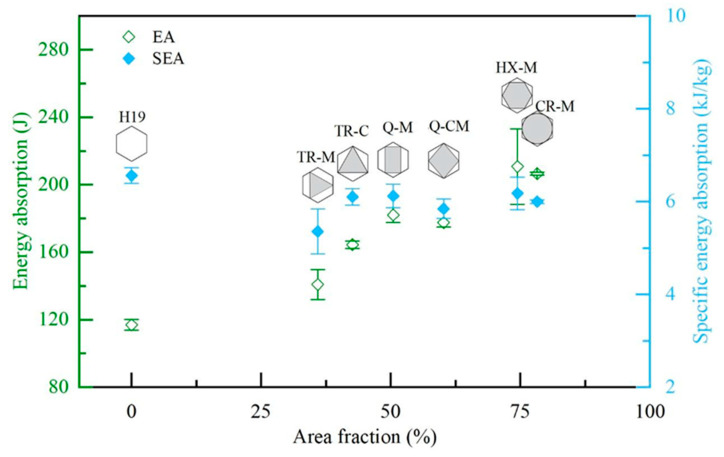
Energy absorption and specific energy absorption as a function of area fraction.

Figure 8 presents energy absorption and specific energy absorption as a function of area fraction. Energy absorption increased with the area fraction. The bare honeycomb had the lowest energy absorption, while the honeycomb with hexagonal fillers showed the highest. Although the CR-M case had a slightly higher area fraction (3.4%) than HX-M, its energy absorption was slightly lower, likely due to lower stress concentration caused by the larger-radius EPS fillers. Additionally, although Q-CM has an area fraction 9.7% higher than the Q-M, with different vertex locations, its energy absorption is 2.5% lower, likely due to the lower densification strain, as depicted in Figure 7.

However, the specific energy absorptions for all partially filled aluminum honeycombs were 18.4% to 5.7% lower than those of the bare honeycomb. Among the cases with foam fillers, specific energy absorption tended to increase with area fraction. The Q-CM case exhibited 6.5% lower specific energy absorption than the Q-M case due to lower energy absorption and higher mass. Similarly, the CR-M case showed 2.8% lower specific energy absorption than HX-M due to lower energy absorption and higher mass. As depicted in Figure 8, the TR-C and Q-M cases exhibit specific energy absorption values close to those of the bare honeycomb H19.

**Figure 9:**
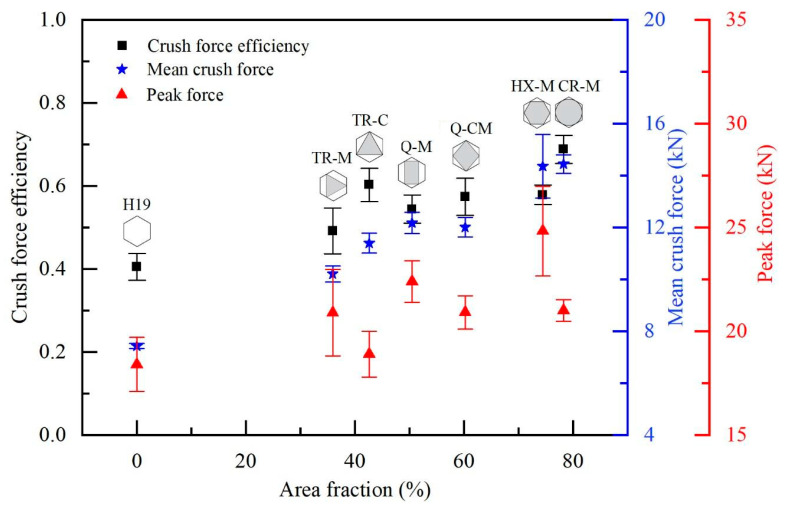
Crush force efficiency, mean crush force, and peak force as a function of area fraction of EPS foam.

Figure 9 depicts the peak force and mean crush force as a function of the area fraction of EPS foam. Comparing the results obtained from TR-M, Q-M, HX-M, and CR-M, of which the vertices were located at the midpoint of the cell walls, both peak force and mean crush force showed an upward trend with increasing area fraction, except the peak force of the CR-M case, which was lower than that of the HX-M despite having a much lower area fraction. This is attributed to the larger radius of circular fillers at the contact area, which reduced stress concentration and consequently lowered the peak force.

When considering the cases with TR-M and TR-C fillers, the mean crush force increased with area fraction, but the peak force did not follow the same trend. Despite the higher area fraction and corner vertex locations in the TR-C case, its peak force was significantly lower than that of the TR-M case. This clearly highlights the impact of vertex locations. The vertex locations of fillers at the cell walls enhance resistance to the compression force during the initial buckling. Similar phenomena were also observed for the cases with Q-M and Q-CM fillers.

Figure 9 also shows the crush force efficiency (*CFE*) as a function of area fraction. The *CFE* increased with the area fraction of the fillers in all cases with the vertex locations at the cell walls, and it was higher than that of the bare honeycomb.

When comparing filled honeycombs with different vertex locations, such as TR-M and TR-C, the TR-C case exhibits significantly higher *CFE* due to its higher mean crush force and lower peak force. A similar trend was observed when comparing Q-M to Q-CM.

### 3.3. Localized Deformation Mechanisms

**Figure 10:**
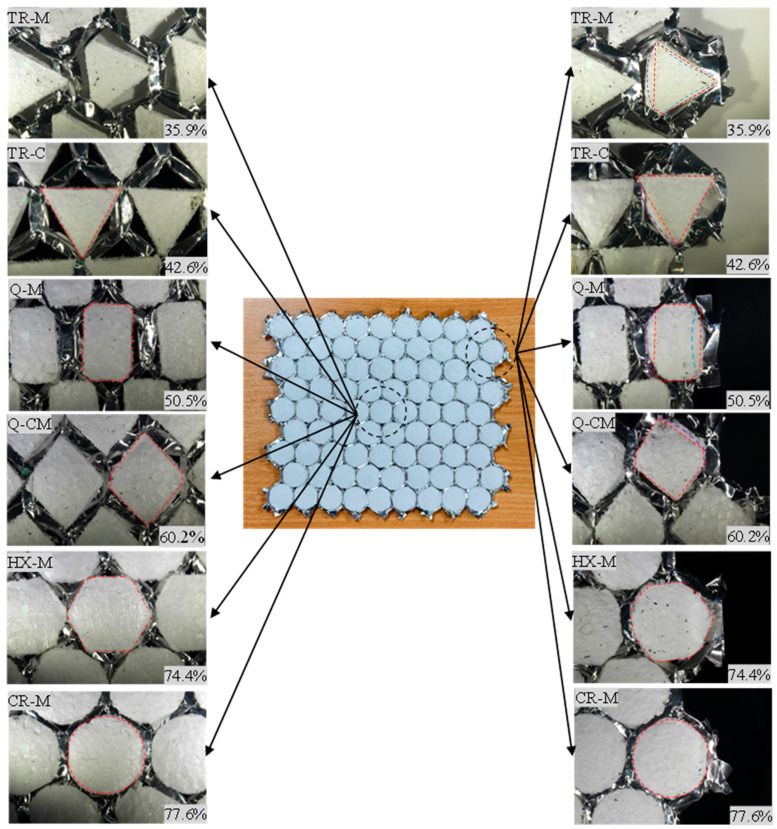
Localized deformation of fillers at center and rim locations.

Figure 10 illustrates the fully deformed, partially foam-filled honeycombs for the TR-M, TR-C, Q-M, Q-CM, HX-M, and CR-M cases at (a) the center and (b) the rim locations. When the walls collapsed, the folded honeycomb walls penetrated the foam filler at the vertices, as illustrated in Figure 10. The degree of penetration varied, with smaller area fractions of the filler exhibiting higher levels of penetration. In contrast, larger area fractions appeared to resist the penetration of the folded walls more effectively. While the penetration effect occurred at both the center and rim locations, it was more pronounced at the rim. This is because the rim has free edges that can deform without any constraint, whereas the cells at the center interact with each other, limiting expansion due to the presence of adjacent cells.

The deformation behavior of the foam filler at the rim location exhibited both inward concavity (blue dotted line) and outward expansion (purple dotted line) at the mid-sections of the filler’s lateral surface along its height for low area fractions. As the area fraction increased, inward concavity became the sole deformation mode observed. The penetration of the honeycomb walls into the foam filler caused the foam material to become trapped between the folded walls, resulting in a lower densification strain.

In addition to the area fraction of the foam filler, which influenced wall penetration, different vertex locations also exhibited varying levels of penetration, as shown in Figure 10 for the TR-M and TR-C cases. During compression, the cell walls collapsed, but in the TR-C filler, where the vertices were positioned at the corners, only a small portion of the foam was penetrated. As a result, the densification strain was higher than in the TR-M case, where a larger portion of the TR-M filler vertices were penetrated by the folded honeycomb walls.

Similarly, in the Q-CM case, which had a higher area fraction than Q-M but different vertex locations, the foam penetration appeared to be less than in the Q-M case. The densification strain was slightly lower. It is noteworthy that two vertices of the Q-CM foam fillers are positioned at double-thickness walls, resulting in increased resistance to collapse.

Based on the above observations, these findings indicate a complex interaction between the honeycomb cell walls and foam fillers during compression, wherein filler deformation is influenced by both vertex location and area fraction.

### 3.4. Discussion and Limitations

The experimental results reveal the significant influence of the foam filler’s area fraction and filler vertex location on the crush characteristics of partially foam-filled honeycombs. These effects arise from the complex interactions between the honeycomb walls and the EPS foam filler, which govern the localized deformation mechanisms and energy absorption performance.

The presence of foam filler delays the onset of buckling by reinforcing the honeycomb walls, thereby extending the elastic zone. Foam fillers in contact with the cell walls act as structural reinforcements, increasing both *P_max_* and *P_m_*. This behavior is particularly pronounced in high-area fraction fillers, where the foam restricts wall deformation more effectively. The delayed buckling onset enhances the material’s structural integrity under moderate loads, making it especially beneficial for applications like crash protection.

The area fraction of the filler also affects wall folding and foam penetration behavior, which are critical to energy absorption performance. Higher area fractions of foam filler demonstrate greater resistance to wall penetration due to increased stiffness. However, greater penetration results in more foam becoming trapped between the folded walls, reducing densification strain and, consequently, lowering energy absorption efficiency. This balance is reflected in the observed trends: as the filler’s area fraction increases, *P_max_*, *P_m_, EA*, *SEA*, and *CFE* also increase.

The geometry of the foam filler further influences crush performance through its impact on the collapse mechanism at the filler–wall interface. Polygon-shaped fillers, such as hexagonal configurations, have small contact areas at their vertices, causing high stress concentrations. In contrast, circular fillers, with their larger contact areas, distribute stress more evenly and reduce stress concentration at the wall. Experimental results show that circular fillers (CR-M), despite their higher area fraction, exhibit lower energy absorption compared to hexagonal fillers (HX-M). This phenomenon arises from the larger radius of circular fillers, which minimizes localized deformation. Conversely, the sharper vertices of hexagonal fillers promote localized deformation and enhance energy absorption. However, the higher peak forces associated with hexagonal fillers reduce their crush force efficiency.

The location of the polygon-shaped filler’s vertices on the filler–wall interface also plays a crucial role in the wall deformation mechanism. When the vertices are positioned at the midpoint of the cell wall (e.g., TR-M and Q-M), the wall is more likely to bend or resist deformation due to the more concentrated force. This results in higher peak forces but reduces the crush force efficiency as the walls do not collapse as uniformly. When located at the corners, however, the stress is more evenly distributed, facilitating a more controlled collapse and, thus, a higher crush force efficiency. Conversely, when vertices are located at the corners (e.g., TR-C and Q-CM), wall collapse becomes more uniform, resulting in improved crush force efficiency. Vertex location also influences foam penetration; fillers with vertices positioned at the corners exhibit smaller penetrated portions compared to those with vertices aligned along the cell walls. This relationship demonstrates that wall penetration significantly contributes to densification strain, which in turn governs energy absorption performance. These findings highlight the critical interplay between filler geometry, area fraction, and vertex location in determining the crush characteristics of partially foam-filled honeycombs. Optimizing these parameters is essential for achieving the desired balance between energy absorption and crush force efficiency, which is vital for applications such as crash protection systems. 

Due to the challenges of precisely cutting irregular filler shapes to control area fraction and vertex locations, conducting parametric studies with a single variable controlled at a time is difficult. Therefore, the direct effect of each parameter cannot be definitively concluded based on the current results. Numerical simulations can be utilized to conduct a parametric study, allowing the isolation and quantification of the individual effects of filler area fraction and vertex location on the mechanical response.

## 4. Conclusions

This study examined the compressive behavior of aluminum honeycombs partially filled with EPS foams with varied filler area fractions, filler shapes, and their vertex contact locations within honeycomb walls. The key findings are as follows:

1. An increase in foam area fraction generally led to higher energy absorption and mean crush force, though with a trade-off in mass and specific energy absorption.
2. Circular-shaped fillers, despite having a higher area fraction than hexagonal ones, displayed lower energy absorption due to their larger radius, which increased the contact area with cell walls and reduced stress concentration.
3. A wall–midpoint contact enhanced a peak force by reinforcing cell walls, while corner contact did not resist force, resulting in low peak force but improved crush force efficiency.
4. The localized deformation mechanism was characterized by the cell wall penetration into the foam filler, which significantly influenced the densification strain. High levels of penetration were observed at lower area fractions, supporting the correlation between increased area fractions and enhanced energy absorption.

These findings highlight the potential of optimized, partially filled honeycomb designs to achieve a balance between weight reduction and enhanced crashworthiness, making them well-suited for lightweight automotive applications.

## Figures and Tables

**Table 1:** EPS foam partially filled Al honeycombs.

Specimen	EPS Foam Filler		Area Fraction
	Cross-Section Shape	Vertex Contact Location	
			(*A.F.*, %)
H19	-	-	0.0
TR-M	Triangular	Wall midpoint	35.91
TR-C	Triangular	Corner	42.63
Q-M	Quadrilateral	Wall midpoint	50.49
Q-CM	Quadrilateral	Corner and wall midpoint	60.18
HX-M	Hexagonal	Wall midpoint	74.43
CR-M	Circular	Wall midpoint	77.63

**Table 2:** Average crush characteristics of filled specimens under axial quasi-static compression tests.

Type	*S_p_* (mm)	*S_d_* (mm)	*P_max_* (kN)	*P_m_* (kN)
H19	0.52	15.8	18.4	7.4
TR-M	1.18	13.6	20.9	10.2
TR-C	1.38	15.6	18.9	11.4
Q-M	1.38	15.0	22.4	12.2
Q-CM	0.90	14.8	20.9	12.0
HX-M	1.48	14.6	24.8	14.3
CR-M	1.02	14.0	20.9	14.4

## Data Availability

The data presented in this study are available on request from the corresponding authors.
